# Association Between Family Structure and Obesity Among Korean Adolescents

**DOI:** 10.2188/jea.JE20250301

**Published:** 2026-09-05

**Authors:** Yoonsoo Sung, Jung Ah Lee

**Affiliations:** Department of Family Medicine, Asan Medical Center, University of Ulsan College of Medicine, Seoul, Republic of Korea

**Keywords:** adolescent, obesity, overweight, family structure, grandparent

## Abstract

**Background:**

In Korea, grandparents often play a crucial role in childrearing. Therefore, this study investigated the association between family structure and obesity among Korean adolescents.

**Methods:**

Data were analyzed from 3,328 adolescents (1,783 male adolescents and 1,545 female adolescents aged 10–18 years) who participated in the Korea National Health and Nutrition Examination Survey from 2018 to 2023. Overweight and obesity were defined as a body mass index ≥85^th^ and ≥95^th^ percentiles, respectively, based on the 2017 Korean Growth Charts. Family structure was categorized as follows: living with both parents; living with a single parent; living with grandparents and parents; or living with grandparents only. Multivariable logistic regression was used to assess associations, adjusting for age and household income, number of siblings, region, parental education level, frequency of eating out, and regular breakfast intake.

**Results:**

The prevalence of obesity was 15.0% (17.7% in male adolescents and 12.1% in female adolescents). Male adolescents living with grandparents and parents were more likely to be overweight (odds ratio [OR] 2.36; 95% confidence interval [CI], 1.46–3.81) or obese (OR 2.47; 95% CI, 1.46–4.19) than those living with both parents, whereas no significant associations with family structure were observed in female adolescents.

**Conclusion:**

Korean male adolescents living with grandparents had higher odds of being overweight or obese. Accordingly, further research is warranted to explore how family structure influences adolescent obesity.

## INTRODUCTION

The prevalence of childhood obesity has increased rapidly, posing a major public health concern worldwide.^1^ In Korea, the prevalence of overweight and obesity among adolescents has risen steadily, nearly doubling from 12.1% in 2009 to 22.3% in 2020.^2^ This upward trend is concerning, as obesity increases the risk of metabolic syndrome. Among children and adolescents with obesity, the prevalence of metabolic syndrome is approximately 26%, which is markedly higher than the 2.8–4.8% reported in the global child and adolescent population.^3^

Childhood obesity develops through a complex interplay of factors, including excessive caloric intake and physical inactivity.^4^ Importantly, children’s dietary habits and physical activity patterns are strongly shaped by family dynamics. Family influences on adolescent obesity extend beyond genetics to include daily routines, emotional relationships, and role-modeling behaviors within the household. Family characteristics, such as emotional support, parental monitoring, and open communication, are associated with healthier dietary behaviors in adolescents.^5^^,^^6^ Similarly, parental modeling, encouragement, and the presence of siblings have been linked to higher physical activity.^7^^–^^9^ Collectively, these findings suggest that both dietary habits and physical activity patterns are deeply embedded within family life.^10^ A wide range of parental factors, including parental weight status, perceptions of a child’s weight, dietary habits, and parenting styles, has been associated with adolescent obesity.^6^^,^^11^ Additionally, family structure, such as living with a single parent, living with grandparents, or having siblings, may influence obesity risk.^12^ In China, a higher prevalence of obesity has been observed among children without siblings.^13^

Although associations between family structure and obesity have been widely examined in Western populations,^12^ their relationship in Asian populations remains underexplored. Family dynamics in Asia differ considerably from those in Western countries, particularly in caregiving practices and household roles.^14^ In Korea, demographic changes, including a sharp rise in dual-income households, have led to shifts in caregiving responsibilities. As of 2021, 45.9% of households with children have two working parents.^15^ Consequently, grandparental involvement in childcare has become increasingly common.^16^ In China, grandparents may contribute to children’s obesity risk through caregiving behaviors, such as feeding practices, snack provision, and supervision routines.^17^ However, large-scale quantitative research on this topic remains limited in East Asian countries, including Korea. Therefore, we aimed to examine the association between family structure and obesity prevalence among Korean adolescents using nationally representative data.

## METHODS

### Study population

This study utilized data from the Korea National Health and Nutrition Examination Survey (KNHANES), a nationally representative, cross-sectional survey conducted by the Korea Disease Control and Prevention Agency since 1998. From 2018 to 2023, a total of 43,745 individuals participated in the survey. Among them, the number of adolescents aged 10–18 years was 701 in 2018, 723 in 2019, 608 in 2020, 588 in 2021, 466 in 2022, and 544 in 2023. After excluding 302 individuals with missing data on family structure or body mass index (BMI), a total of 3,328 adolescents were included in the final analysis.

### Demographic characteristics and family structure

The KNHANES selected households based on geographic region and conducted individual-level surveys within the sampled households. In this survey, the regions were classified into 17 metropolitan areas and provinces in South Korea. Furthermore, participants were classified as living in rural or urban areas based on their administrative address (‘-dong’ for urban and ‘-eup’ or ‘-myeon’ for rural) as recorded in the KNHANES. Demographic information was collected through standardized face-to-face interviews with questionnaires developed by the Korea Disease Control and Prevention Agency.^18^ Household income was evaluated using equivalized monthly gross household income, calculated as the total monthly household income divided by the square root of the number of household members. Parental education level was determined by linking each child’s record to parent records using the identifiers of father and mother and was categorized as follows: elementary school graduate, middle school graduate, high school graduate, and college graduate or higher. The “unknown” parental education category included cases in which information could not be retrieved because the record was unavailable (eg, when a parent did not participate in the survey or the education variable was missing) or because the parent refused to respond.

Lifestyle behaviors were assessed using self-reported questionnaires. Physical activity was evaluated among adolescents aged 12 years and older by asking them how many days in the past week they engaged in at least 60 minutes of exercise. Adolescents who exercised at least once per week were classified as physically active, whereas those who exercised less than once per week were classified as inactive.

A dietary survey was conducted using a questionnaire among a subset of participants (*N* = 2,895; 87.0%) who completed the Dietary Survey component of the KNHANES, which assessed regular breakfast consumption and frequency of eating out. Regular breakfast consumption was defined as eating breakfast at least three times per week over the past year. Those who ate breakfast two times or fewer per week were categorized as not having a regular breakfast. The frequency of eating out was assessed by asking the following question: “During the past year, on average, how often did you consume meals prepared outside the home (including eating out, delivery food, take-out, institutional meals, or meals provided by religious organizations)?” For analysis, response options were categorized into three groups: “at least once daily,” “three to six times weekly,” or “two times or fewer per month.”

Family structure was identified by asking the following question: “Which of the following best describes your household type?” Response options were as follows: 1) single household; 2) couple without children; 3) couple with unmarried children; 4) single parent with unmarried children; or 5) multigenerational household (three or more generations). To further classify family structure in relation to grandparental cohabitation, the following two additional questions were asked: “How many people live in your household?” and “What is your relationship with the primary household member?” Based on these responses, family structure was categorized into the following four groups: (1) living with both parents; (2) living with a single parent; (3) living with grandparents only; and (4) living with grandparents and parents (either both parents or a single parent). The presence of siblings was also determined using the same set of household questions.

### Anthropometric measures and outcome variables

Body weight was measured using a standardized protocol with participants wearing light examination gowns and no shoes. Height and weight were measured to the nearest 0.1 cm and 0.1 kg, respectively. Waist circumference was measured in the standing position, with the upper garment lifted and secured at the midpoint between the lower margin of the last rib and the upper margin of the iliac crest, to an accuracy of 0.1 cm. BMI was calculated as weight (kg) divided by height squared (m^2^). The primary outcomes were overweight and obesity, defined according to age- and sex-specific BMI percentiles based on the 2017 Korean Growth Charts.^19^ Overweight and obesity were defined as BMI ≥85^th^ percentile and ≥95^th^ percentile, respectively.

Blood pressure was measured by trained nurses using a standardized protocol with a calibrated sphygmomanometer, in accordance with the national quality-control program of KNHANES. Three consecutive measurements were obtained, and the average value was used for analysis. Blood samples were collected after overnight fasting and analyzed in certified laboratories. Biochemical parameters, including glucose and lipid profiles, were measured using enzymatic methods, with continuous internal and external quality assurance programs to ensure reliability.

### Statistical analysis

All statistical analyses incorporated sampling weights and accounted for the complex survey design to ensure national representativeness. Descriptive statistics are presented as unweighted sample counts and weighted percentages. Categorical variables were compared using the chi-square test or Fisher’s exact test, as appropriate, based on overweight and obesity status. The prevalence of underweight, overweight, and obesity according to family structure was presented as weighted percentages using chi-square tests. Logistic regression models were used to estimate odds ratios (ORs) and 95% confidence intervals (CIs) for overweight and obesity according to family structure. Analysis of all models was performed separately for male and female adolescents owing to differences in growth percentiles between sexes. Model 1 was adjusted for age and survey year; model 2 was further adjusted for socioeconomic status; and model 3 was additionally adjusted for dietary habits. Because physical activity was assessed only among adolescents aged 12 years and older, model 3 results are presented in eTable 1 with an additional adjustment for physical activity in this subgroup. Furthermore, additional analyses were conducted by dividing the age groups into 10–14 years (late elementary or middle school) and 15–18 years (late middle school or high school). Missing values, which accounted for less than 5% of the total adolescents, were excluded. The dietary survey was conducted among participants who provided consent, with adjusted weights applied in the analysis. Accordingly, dietary habits that were not collected because of the study design were not treated as missing. All statistical analyses were performed using Stata, version 19.0 (StataCorp LLC, College Station, TX, USA). A *P*-value of <0.05 was considered statistically significant.

## RESULTS

Table 1 presents the demographic characteristics of the study population (*N* = 3,328). The mean age of the participants was 13.6 (standard deviation [SD], 2.6) years. Male adolescents had higher mean BMI (21.7; SD, 4.4 vs 20.3; SD, 3.8 kg/m^2^), waist circumference (74.2; SD, 12.0 vs 67.5; SD, 9.0 cm), systolic blood pressure (110.5; SD, 10.2 vs 105.0; SD, 8.7 mm Hg), and fasting glucose levels (93.0; SD, 7.3 vs 91.5; SD, 10.3 mg/dL) than female adolescents. Conversely, female adolescents had higher mean levels of total cholesterol (161.8; SD, 27.5 vs 166.4; SD, 26.8 mg/dL) and triglycerides (87.8; SD, 52.7 vs 89.8; SD, 48.5 mg/dL). In general, parental education level was college graduate or higher (42.2% for fathers and 55.1% for mothers). Regarding family structure, 74.8% of participants lived with both parents, 16.4% with a single parent, 1.3% with grandparents only, and 7.6% with grandparents together with one or both parents. Approximately 40.8% of participants had no siblings, 49.4% had one sibling, and 9.8% had two or more siblings. Notably, male adolescents had a higher prevalence of obesity (17.7% vs 12.1%) and overweight (10.9% vs 8.1%) than female adolescents.

**Table 1.** Demographic characteristics of study participants (*N* = 3,328)

	Total (*n* = 3,328)	Males (*n* = 1,783)	Females (*n* = 1,545)
	Mean (SD)		

Age, years	13.6 (2.6)	13.6 (2.6)	13.6 (2.6)
BMI, kg/m^2^	21.1 (4.2)	21.7 (4.4)	20.3 (3.8)
Waist circumference, cm	71.1 (11.2)	74.2 (12.0)	67.5 (9.0)
Systolic blood pressure, mm Hg	108.0 (10.0)	110.5 (10.2)	105.0 (8.7)
Diastolic blood pressure, mm Hg	65.9 (8.6)	66.0 (9.2)	65.8 (7.9)
Hemoglobin, g/dL	14.0 (1.3)	14.6 (1.1)	13.2 (1.0)
Total cholesterol, mg/dL	163.9 (27.3)	161.8 (27.5)	166.4 (26.8)
Fasting glucose, mg/dL	92.3 (8.9)	93.0 (7.3)	91.5 (10.3)
Triglycerides, mg/dL	88.8 (50.8)	87.8 (52.7)	89.8 (48.5)
Total calorie intake, kcal	1,982.6 (809.1)	2,186.6 (856.9)	1,746.5 (677.9)

	*N* (%)		

**Year**			
2018	630 (17.8)	335 (17.3)	295 (18.2)
2019	656 (17.3)	354 (17.4)	302 (17.2)
2020	553 (16.2)	313 (16.7)	240 (15.7)
2021	511 (16.1)	271 (16.0)	240 (16.3)
2022	453 (16.0)	246 (16.2)	207 (15.8)
2023	525 (16.6)	264 (16.4)	261 (16.7)
**Household income**			
1^st^ quartile	259 (7.7)	133 (7.5)	126 (7.9)
2^nd^ quartile	859 (25.7)	456 (25.6)	403 (25.8)
3^rd^ quartile	1,164 (35.5)	626 (35.5)	538 (35.2)
4^th^ quartile	1,037 (31.1)	563 (31.2)	474 (30.8)
**Paternal education level**			
Elementary school	25 (0.6)	10 (0.4)	15 (0.8)
Middle school	72 (2.1)	43 (2.3)	29 (1.8)
High school	711 (21.0)	403 (23.3)	308 (18.4)
College degree or higher	1,428 (42.2)	781 (42.8)	647 (41.6)
Unknown^a^	1,092 (34.2)	546 (31.2)	546 (37.5)
**Maternal education level**			
Elementary school	34 (1.0)	14 (0.7)	20 (1.3)
Middle school	66 (1.9)	33 (1.8)	33 (1.9)
High school	1,050 (32.1)	572 (32.6)	478 (31.7)
College degree or higher	1,844 (55.1)	973 (54.0)	871 (56.3)
Unknown^a^	334 (9.9)	191 (10.9)	143 (10.8)
**Region**			
Rural	527 (13.7)	268 (12.9)	259 (14.6)
Urban	2,801 (86.3)	1,515 (87.1)	1,286 (85.4)
**Regular breakfast**			
No	833 (26.1)	433 (25.9)	400 (26.3)
Yes	2,062 (60.4)	1,120 (60.6)	942 (60.3)
Not applicable^b^	433 (13.5)	230 (13.6)	203 (13.5)
**Frequency of eating out**			
At least once daily	1,133 (34.5)	608 (34.8)	525 (34.2)
Three to six times weekly	1,612 (46.9)	870 (46.7)	742 (47.1)
Two times or fewer per week	150 (5.1)	75 (5.0)	75 (5.3)
Not applicable^b^	433 (13.5)	230 (13.6)	203 (13.5)
**Family structure**			
Living with both parents	2,492 (74.8)	1,366 (77.0)	1,126 (74.8)
Living with single parent	526 (16.4)	259 (14.7)	267 (18.2)
Living with only grandparents	42 (1.3)	29 (1.7)	13 (0.8)
Living with grandparents and parents	268 (7.6)	129 (6.6)	139 (8.7)
**Number of siblings**			
0	1,311 (40.8)	711 (41.9)	600 (39.6)
1	1,673 (49.4)	921 (50.2)	752 (48.6)
2	344 (9.8)	151 (7.9)	193 (11.8)
**Categories of BMI**			
Underweight	256 (7.6)	125 (6.8)	131 (8.5)
Normal	2,248 (67.8)	1,153 (64.7)	1,095 (71.3)
Overweight	340 (9.6)	203 (10.9)	137 (8.1)
Obesity	484 (15.0)	302 (17.7)	182 (12.1)

BMI, body mass index; *N*, number of participants; SD, standard deviation.

^a^Information on parental education was unavailable because the parental record could not be retrieved (eg, the parent did not participate in the survey, or the education variable was missing) or because the parent refused to respond.

^b^Participants not included in the dietary survey.

Table 2 presents the prevalence of underweight, overweight, and obesity stratified by survey year, age, sociodemographic factors, and lifestyle factors. Among male adolescents, obesity prevalence was highest among those whose fathers had completed high school (24.8%), whereas among female adolescents, it was highest among those whose fathers had completed middle school (21.6%). Maternal education was also associated with obesity in female adolescents, with the lowest prevalence observed when mothers had attained a college degree (8.8%). Additionally, family structure was strongly associated with obesity in male adolescents, with those living with both parents and grandparents exhibiting a substantially higher prevalence (34.1%).

**Table 2.** Prevalence of underweight, overweight, and obesity according to participant characteristics

	Males, weighted %					Females, weighted %				

	Underweight	Normal	Overweight	Obesity	*P* value	Underweight	Normal	Overweight	Obesity	*P* value
**Year**					0.417					0.412
2018	8.50	65.39	10.01	16.09		6.87	76.23	7.57	9.33	
2019	7.30	66.79	10.96	14.95		10.34	65.75	9.86	14.05	
2020	6.67	62.72	12.56	18.05		9.98	73.64	5.64	10.73	
2021	5.68	60.53	8.66	25.13		9.56	69.47	8.22	12.75	
2022	5.77	63.65	13.07	17.51		6.52	71.21	11.42	10.85	
2023	6.57	68.99	9.82	14.63		7.99	71.04	6.13	14.84	
**Household income**					0.572					0.575
1^st^ quartile	4.06	68.38	13.52	14.05		6.97	66.92	10.24	15.87	
2^nd^ quartile	6.30	65.14	9.02	19.54		8.26	68.02	9.91	13.80	
3^rd^ quartile	7.32	63.67	10.09	18.91		9.15	72.67	7.77	10.41	
4^th^ quartile	7.27	64.83	12.36	15.54		8.53	73.67	6.61	11.19	
**Paternal education level**					0.038					0.038
Elementary school	9.61	78.71	3.20	8.49		7.11	47.46	30.30	15.13	
Middle school	10.69	58.72	9.70	20.90		8.08	59.91	10.37	21.64	
High school	5.51	61.77	7.88	24.84		5.51	71.27	8.95	14.27	
College degree or higher	7.55	66.03	10.95	15.47		11.04	70.75	7.93	10.28	
Unknown^a^	6.33	65.38	13.12	15.17		7.27	72.86	7.39	12.49	
**Maternal education level**					0.122					0.003
Elementary school	20.32	52.24	6.68	20.75		2.29	53.44	21.06	23.22	
Middle school	12.32	56.83	3.97	26.88		2.78	63.08	8.52	25.62	
High school	6.25	64.14	9.49	20.12		8.47	66.04	9.91	15.58	
College degree or higher	7.01	66.75	11.63	14.61		8.78	75.10	7.27	8.84	
Unknown^a^	5.42	58.51	12.48	23.60		9.25	69.75	5.31	15.69	
**Region**					0.451					0.015
Rural	4.80	62.81	11.19	21.21		7.61	63.49	10.66	18.24	
Urban	7.07	65.00	10.80	17.13		8.69	72.58	7.71	11.03	
**Regular breakfast**					0.997					0.081
No	7.13	63.68	11.36	17.83		6.84	68.32	8.59	16.25	
Yes	6.77	64.77	10.81	17.64		9.50	71.05	8.23	11.22	
Not applicable^b^	6.09	66.51	10.05	17.34		7.46	77.86	6.85	7.84	
**Frequency of eating out**					0.552					0.062
At least once daily	7.72	64.90	12.09	15.29		8.97	70.71	7.54	12.78	
Three to six times weekly	6.72	64.25	10.39	18.64		7.57	70.14	8.66	13.63	
Two times or fewer per week	2.53	62.98	8.69	25.80		16.91	67.82	10.57	4.70	
Not applicable^b^	6.09	66.51	10.05	17.34		7.46	77.86	6.85	7.84	
**Family structure**					<0.001					0.382
Living with both parents	7.60	66.02	10.06	16.33		8.37	72.32	7.14	12.17	
Living with single parent	3.98	67.06	12.97	16.00		7.70	71.17	9.85	11.28	
Living with only grandparents	5.02	43.94	23.18	27.86		—	72.03	8.13	19.84	
Living with grandparents and parents	3.87	49.70	12.29	34.14		12.36	62.41	12.88	12.35	
**Presence of siblings**					0.675					0.145
0	6.94	63.82	11.70	17.54		8.95	70.01	8.67	12.37	
1	6.50	64.45	10.48	18.58		8.19	73.78	6.41	11.62	
2	7.66	71.19	8.77	12.38		8.51	64.98	13.47	13.04	

^a^Information on parental education was unavailable because the parental record could not be retrieved (eg, the parent did not participate in the survey, or the education variable was missing) or because the parent refused to respond.

^b^Participants not included in the dietary survey.

Table 3 presents the results of the univariable logistic regression analysis of overweight and obesity. Among male adolescents, no clear associations were observed between overweight or obesity and parental education, household income, region, or number of siblings. However, the odds of being overweight were higher among female adolescents whose fathers (OR 3.74; 95% CI, 1.06–13.21) and mothers (OR 4.14; 95% CI, 1.31–13.05) were elementary school graduates compared with those whose parents were college graduates. A regular breakfast habit was associated with lower odds of obesity (OR 0.65; 95% CI, 0.44–0.97) in female adolescents. Family structure was only significant in male adolescents, with those living with grandparents and parents having higher odds of being overweight (OR 2.42; 95% CI, 1.50–3.90) and obesity (OR 2.66; 95% CI, 1.53–4.62) than those living with both parents. The year of survey showed no clear association in either sex.

**Table 3.** Univariable logistic regression analysis of overweight and obesity according to participant characteristics

	Males				Females			

	BMI ≥85^th^		BMI ≥95^th^		BMI ≥85^th^		BMI ≥95^th^	

	OR (95% CI)	*P* value	OR (95% CI)	*P* value	OR (95% CI)	*P* value	OR (95% CI)	*P* value
**Age, 1 year increase**	0.97 (0.92–1.01)	0.170	1.01 (0.96–1.07)	0.669	1.05 (0.99–1.10)	0.104	1.07 (1.00–1.15)	0.042
**Year**								
2018	1		1		1		1	
2019	0.99 (0.64–1.64)	0.965	0.92 (0.56–1.51)	0.733	1.55 (0.96–2.47)	0.070	1.59 (0.88–2.86)	0.123
2020	1.25 (0.84–1.86)	0.273	1.15 (0.73–1.82)	0.551	0.96 (0.60–1.55)	0.876	1.17 (0.64–2.13)	0.612
2021	1.44 (0.95–2.19)	0.084	1.75 (1.10–2.80)	0.019	1.30 (0.80–2.14)	0.291	1.42 (0.77–2.61)	0.259
2022	1.25 (0.82–1.91)	0.306	1.11 (0.68–1.82)	0.686	1.41 (0.86–2.31)	0.173	1.18 (0.62–2.27)	0.615
2023	0.92 (0.58–1.46)	0.711	0.89 (0.52–1.53)	0.680	1.30 (0.83–2.05)	0.249	1.69 (0.94–3.04)	0.078
**Household income**								
1^st^ quartile	1		1		1		1	
2^nd^ quartile	1.05 (0.62–1.77)	0.852	1.49 (0.81–2.71)	0.196	0.88 (0.52–1.48)	0.627	0.85 (0.45–1.61)	0.614
3^rd^ quartile	1.07 (0.65–1.76)	0.778	1.43 (0.81–2.50)	0.214	0.63 (0.37–1.08)	0.090	0.62 (0.32–1.19)	0.148
4^th^ quartile	1.02 (0.62–1.66)	0.947	1.13 (0.62–2.06)	0.700	0.61 (0.36–1.03)	0.064	0.67 (0.34–1.30)	0.234
**Paternal education level**								
Elementary school	0.37 (0.06–2.22)	0.276	0.51 (0.06–4.47)	0.540	3.74 (1.06–13.21)	0.041	1.56 (0.36–6.74)	0.554
Middle school	1.23 (0.52–2.90)	0.639	1.44 (0.56–3.70)	0.445	2.11 (0.81–5.49)	0.124	2.41 (0.79–7.33)	0.121
High school	1.35 (0.98–1.87)	0.066	1.81 (1.23–2.64)	0.002	1.36 (0.94–1.96)	0.100	1.45 (0.93–2.26)	0.100
College degree or higher	1		1		1		1	
Unknown^a^	1.10 (0.83–1.45)	0.505	0.98 (0.69–1.39)	0.898	1.11 (0.81–1.54)	0.513	1.24 (0.83–1.88)	0.295
**Maternal education level**								
Elementary school	1.06 (0.32–3.48)	0.920	1.53 (0.38–6.15)	0.548	4.14 (1.31–13.05)	0.016	3.12 (0.76–12.80)	0.114
Middle school	1.25 (0.51–3.07)	0.620	2.15 (0.83–5.58)	0.116	2.70 (1.13–6.44)	0.025	3.55 (1.42–8.86)	0.007
High school	1.18 (0.90–1.56)	0.233	1.47 (1.07–2.03)	0.018	1.78 (1.31–2.42)	<0.001	1.90 (1.28–2.83)	0.002
College degree or higher	1		1		1		1	
Unknown^a^	1.59 (1.01–2.50)	0.047	1.81 (1.06–3.08)	0.030	1.38 (0.85–2.26)	0.195	1.92 (1.07–3.46)	0.030
**Region**								
Rural	1		1		1		1	
Urban	0.81 (0.57–1.14)	0.223	0.77 (0.51–1.14)	0.194	0.57 (0.39–0.83)	0.004	0.56 (0.36–0.87)	0.010
**Regular breakfast**								
No	1		1		1		1	
Yes	0.97 (0.71–1.31)	0.821	0.99 (0.68–1.42)	0.946	0.73 (0.53–1.02)	0.062	0.65 (0.44–0.97)	0.037
Not applicable^b^	0.92 (0.60–1.39)	0.681	0.97 (0.59–1.58)	0.894	0.52 (0.33–0.83)	0.006	0.44 (0.24–0.82)	0.009
**Frequency of eating out**								
At least once daily	1		1		1		1	
Three to six times weekly	1.08 (0.83–1.42)	0.556	1.27 (0.91–1.77)	0.159	1.12 (0.83–1.52)	0.445	1.07 (0.73–1.58)	0.708
Two times or fewer per week	1.40 (0.72–2.70)	0.320	1.93 (0.88–4.21)	0.101	0.71 (0.38–1.33)	0.281	0.34 (0.13–0.86)	0.022
Not applicable^b^	1.00 (0.68–1.47)	0.996	1.16 (0.73–1.85)	0.525	0.67 (0.43–1.05)	0.083	0.58 (0.32–1.06)	0.075
**Family structure**								
Living with both parents	1		1		1		1	
Living with single parent	1.14 (0.81–1.59)	0.452	0.98 (0.63–1.51)	0.913	1.12 (0.77–1.63)	0.554	0.92 (0.55–1.54)	0.743
Living with only grandparents	2.91 (1.22–6.93)	0.016	1.98 (0.78–5.03)	0.151	1.62 (0.45–5.91)	0.462	1.79 (0.47–6.80)	0.394
Living with grandparents and parents	2.42 (1.50–3.90)	<0.001	2.66 (1.53–4.62)	0.001	1.41 (0.87–2.30)	0.167	1.01 (0.54–1.93)	0.959
**Presence of siblings**								
0	1		1		1		1	
1	0.99 (0.76–1.29)	0.948	1.07 (0.79–1.47)	0.658	0.82 (0.62–1.10)	0.196	0.93 (0.65–1.33)	0.698
2	0.65 (0.39–1.08)	0.098	0.66 (0.36–1.21)	0.182	1.35 (0.88–2.08)	0.164	1.06 (0.61–1.85)	0.831

BMI, body mass index; CI, confidence interval; OR, odds ratio.

^a^Information on parental education was unavailable because the parental record could not be retrieved (eg, the parent did not participate in the survey, or the education variable was missing) or because the parent refused to respond.

^b^Participants not included in the dietary survey.

Table 4 presents the results of the multivariable logistic regression analysis for overweight and obesity according to family structure. Among male adolescents, those living with grandparents and parents had significantly higher odds of being overweight (OR 2.36; 95% CI, 1.46–3.81) and obesity (OR 2.47; 95% CI, 1.46–4.19) across all models, even after adjustment for sociodemographic and lifestyle factors. Living with grandparents only was also associated with increased odds of being overweight in male adolescents (OR 3.87; 95% CI, 1.23–12.18). In contrast, no significant associations between family structure and overweight or obesity were observed among female adolescents. To adjust for physical activity, the analysis was conducted among adolescents aged 12 years and older, and the results remained consistent (eTable 1). Given that the influence of caregivers may be smaller in older adolescents, further age-stratified analyses were conducted, revealing that male adolescents living with grandparents had increased odds of obesity (eTable 2 and eTable 3).

**Table 4.** Multivariable logistic regression analysis of overweight and obesity according to family structure

			BMI ≥85^th^		BMI ≥95^th^	

			OR (95% CI)	*P* value	OR (95% CI)	*P* value
Males	Model 1	Both parents	1		1	
		Single parent	1.16 (0.82–1.64)	0.407	0.94 (0.60–1.48)	0.804
		Only grandparents	3.25 (1.34–7.89)	0.009	2.06 (0.80–5.34)	0.136
		Grandparents and parents	2.49 (1.55–3.99)	<0.001	2.76 (1.63–4.69)	<0.001
	Model 2	Both parents	1		1	
		Single parent	1.35 (0.87–2.09)	0.176	1.25 (0.71–2.18)	0.440
		Only grandparents	3.77 (1.32–10.75)	0.013	3.01 (0.94–9.62)	0.063
		Grandparents and parents	2.50 (1.57–3.99)	<0.001	2.66 (1.58–4.49)	<0.001
	Model 3	Both parents	1		1	
		Single parent	1.41 (0.86–2.29)	0.171	1.44 (0.78–2.66)	0.242
		Only grandparents	3.87 (1.23–12.18)	0.021	3.50 (0.95–12.91)	0.060
		Grandparents and parents	2.36 (1.46–3.81)	<0.001	2.47 (1.46–4.19)	0.001

Females	Model 1	Both parents	1		1	
		Single parent	1.07 (0.73–1.57)	0.717	0.85 (0.50–1.43)	0.537
		Only grandparents	1.70 (0.43–6.68)	0.446	1.98 (0.50–7.89)	0.333
		Grandparents and parents	1.43 (0.88–2.34)	0.153	1.00 (0.54–1.87)	0.995
	Model 2	Both parents	1		1	
		Single parent	1.10 (0.68–1.79)	0.687	0.70 (0.38–1.27)	0.237
		Only grandparents	2.19 (0.48–9.93)	0.307	1.37 (0.28–6.74)	0.686
		Grandparents and parents	1.38 (0.82–2.33)	0.224	0.88 (0.45–1.72)	0.703
	Model 3	Both parents	1		1	
		Single parent	1.08 (0.65–1.80)	0.767	0.67 (0.35–1.28)	0.224
		Only grandparents	2.46 (0.49–12.30)	0.273	1.48 (0.27–8.20)	0.655
		Grandparents and parents	1.28 (0.73–2.26)	0.392	0.82 (0.40–1.70)	0.598

BMI, body mass index; CI, confidence interval; OR, odds ratio.

Model 1: Adjusted for age and year.

Model 2: Adjusted for age, year, region, household income, parental education level, and the number of siblings.

Model 3: Adjusted for age, year, region, household income, parental education level, and the number of siblings, regular breakfast habits and frequency of eating out.

Family structure categories indicate participants’ living arrangements (ie, living with both parents, a single parent, only grandparents, or grandparents and parents).

## DISCUSSION

In this study, adolescents living with grandparents were substantially more likely than those living with both parents to be overweight or obese. This result was consistent even after adjusting for age, household income, parental education, number of siblings, and dietary habits. This association was particularly pronounced among male adolescents, whereas no notable association was observed among female adolescents.

A major strength of this study is the use of representative national data from Korea and the reliance on directly measured BMI rather than self-reported values,^17^ which improves the accuracy of obesity classification. Participants were categorized into four distinct family structures—living with both parents, a single parent, grandparents and parents, or grandparents only—allowing for a more detailed analysis. Additionally, the models were adjusted for key confounding variables, such as socioeconomic status and dietary habits. Overweight and obesity were classified using the 85^th^ and 95^th^ percentiles of the 2017 Korean National Growth Chart,^19^ thereby providing a more detailed understanding of weight status. Importantly, the analysis was stratified according to sex and age groups to account for sex-specific and developmental differences among adolescents. The lack of association in female participants may reflect stronger sociocultural pressures regarding body image, which are more prevalent among female adolescents and may influence eating and exercise behaviors.^20^^–^^22^

The study findings are consistent with previous research suggesting that grandparental involvement can contribute to childhood obesity. Similar results were reported in Japan, where children cared for by grandparents in early childhood had a higher risk of between-meal eating, higher mean BMI in both sexes, and a sustained excess prevalence of overweight among male adolescents up to early adulthood.^23^ Grandparents may overfeed children, provide energy-dense snacks, and restrict physical activity, often due to traditional caregiving beliefs.^16^^,^^17^ These behaviors have been observed in China and other cultural contexts.^12^^,^^16^^,^^17^ Grandparents may associate larger body size with health and well-being and engage in more permissive feeding practices.^12^^,^^13^^,^^17^ Such patterns likely reflect generational differences in health knowledge and cultural norms. The elevated odds of being overweight and obese observed in adolescents living with grandparents in this study reinforce the need to consider the role of grandparents in public health strategies targeting children. Moreover, living with grandparents only was associated with being overweight, but not clearly with obesity. This may be attributed to differences in socioeconomic status between children and adolescents living with grandparents only and those living with both parents and grandparents. In particular, previous research has reported that households headed by grandparents tend to have higher rates of poverty rates.^24^ Therefore, it seems more appropriate to compare adolescents living with both parents and those living with both parents and grandparents to examine the influence of grandparental caregiving, rather than focusing on children living with grandparents only.

Although previous studies have suggested that having siblings may reduce the risk of obesity by encouraging more physical activity or reducing parental overfeeding,^13^ no notable association was observed between sibling presence and overweight or obesity in the current study. This may be partly explained by Korea’s low fertility rate, which is among the lowest in the world,^25^^,^^26^ potentially reducing the statistical power to detect such an association. Specifically, the adolescents in this study were born between 2003 and 2013, a period during which South Korea’s fertility rate experienced a pronounced decline. The fertility rate fell from 1.23 in 2005 to 0.92 in 2015, indicating a substantial decrease in the number of children born per woman.^26^ Considering that associations between household income and adolescent overweight and obesity have been reported in Korea,^27^^–^^29^ household income was adjusted for in multivariable analysis. Although not statistically significant in this study, a tendency for lower prevalence of overweight with higher household income was observed among girls. Notably, higher paternal and maternal education levels were associated with a lower prevalence of overweight among female participants. Parental education influences children’s weight,^27^^,^^28^ and obesity among adolescents is more strongly associated with parental education than with household income. Consistent with prior Korean studies,^27^^,^^28^ the present findings also suggest that parental education may have a greater association with adolescent weight status than household income.

Contrary to findings from Western countries—where parental separation or divorce is often linked to disrupted routines, reduced supervision, and increased psychosocial stress, all of which may negatively impact children’s health behaviors^12^^,^^30^—this study did not find a notable association between single-parent households and adolescent overweight or obesity in Korea. Cultural and familial contexts may help to explain this difference. In Korea, the proportion of single-parent households remains relatively low, and caregiving responsibilities are frequently shared with extended family, including grandparents.^31^ Furthermore, legal and caregiving structures differ across countries. In many Western countries, post-divorce childcare often involves non-custodial parents.^32^ In contrast, in Korea and Japan, visitation rights are less rigorously enforced, and children are more likely to be solely cared for by one parent.^33^^,^^34^ These structural and cultural differences may contribute to the absence of an association between single parenthood and adolescent obesity in the Korean study sample.

This study has several limitations. First, its cross-sectional design precludes causal inference regarding the relationship between family structure and adolescent obesity. Second, although family structure was classified based on household composition, this may not fully capture caregiving roles or quality, particularly in cases where non-residential grandparents are involved. Third, unmeasured confounders, such as parenting style, emotional support, genetic predisposition, or parental BMI, may have influenced the results. Although this study focused on family structure rather than parental characteristics, future research should consider incorporating these variables to provide a more comprehensive understanding. Additionally, the “Not applicable” category in dietary variables included participants who did not complete the dietary survey. Therefore, associations with overweight and obesity should be interpreted cautiously, as these findings may reflect differences in characteristics between survey participants and nonparticipants. Despite these limitations, this study offers meaningful insights into the association between family structure and adolescent obesity among Korean adolescents. By using representative national data and adjusting for relevant sociodemographic and behavioral factors, this study highlights the potential impact of non-traditional caregiving arrangements, particularly grandparental care, on adolescent weight status. These findings underscore the importance of family context in shaping adolescent health and support the development of culturally tailored public health interventions.

In conclusion, the findings of this study highlight the substantial role of family structure in adolescent obesity, particularly the increased risk observed among male adolescents living with grandparents. Overall, these findings underscore the importance of incorporating extended caregiving arrangements into the design of family-based public health interventions in Korea.

## SUPPLEMENTARY MATERIAL

eTable 1. Multivariable logistic regression analysis of overweight and obesity according to family structure (age 12–18 years)

eTable 2. Multivariable logistic regression analysis of overweight and obesity according to family structure among male adolescents

eTable 3. Multivariable logistic regression analysis of overweight and obesity according to family structure among female adolescents
