# Supplementary material for: Association Between Family Structure and Obesity Among Korean Adolescents
Source: J Epidemiol. 2026 Sep 5;36(9):281–8. doi: 10.2188/jea.JE20250301 (PMC13447040; doi:10.2188/jea.JE20250301)
Supplement: Supplementary file 1 [file je-36-281-s001.pdf]

eTable 1. Multivariable logistic regression analysis of overweight and obesity according to family structure (age 12–18 years)

			BMI ≥85th		BMI ≥95th	
			OR (95% CI)	*P* value	OR (95% CI)	*P* value
Males	Model 1	Both parents	1		1	
		Single parent	1.21 (0.82–1.79)	0.329	1.05 (0.64–1.73)	0.848
		Only grandparents	3.43 (1.29–9.16)	0.014	2.37 (0.82–6.89)	0.112
		Grandparents and parents	2.40 (1.35–4.27)	0.003	3.23 (1.76–5.93)	0.000
	Model 2	Both parents	1		1	
		Single parent	1.41 (0.85–2.31)	0.180	1.43 (0.77–2.67)	0.259
		Only grandparents	3.74 (1.13–12.32)	0.030	3.63 (0.98–13.50)	0.055
		Grandparents and parents	2.33 (1.33–4.07)	0.003	3.01 (1.67–5.43)	0.000
	Model 3	Both parents	1		1	
		Single parent	1.39 (0.79–2.43)	0.249	1.72 (0.85–3.48)	0.133
		Only grandparents	3.92 (1.11–13.89)	0.031	3.96 (0.92–17.01)	0.064
		Grandparents and parents	2.10 (1.17–3.77)	0.013	2.72 (1.48–5.01)	0.001
Females	Model 1	Both parents	1		1	
		Single parent	1.11 (0.72–1.72)	0.630	0.96 (0.54–1.68)	0.877
		Only grandparents	2.18 (0.49–9.64)	0.306	2.47 (0.56–10.99)	0.234
		Grandparents and parents	1.47 (0.81–2.65)	0.205	1.08 (0.51–2.28)	0.848
	Model 2	Both parents	1		1	
		Single parent	1.19 (0.69–2.07)	0.547	0.81 (0.42–1.57)	0.533
		Only grandparents	2.65 (0.50–14.06)	0.260	1.77 (0.32–9.77)	0.510
		Grandparents and parents	1.47 (0.78–2.77)	0.237	0.98 (0.43–2.23)	0.953
	Model 3	Both parents	1		1	
		Single parent	1.20 (0.65–2.21)	0.567	0.79 (0.38–1.65)	0.537
		Only grandparents	4.07 (0.62–26.53)	0.142	2.79 (0.40–19.73)	0.303
		Grandparents and parents	1.39 (0.69–2.80)	0.350	0.96 (0.39–2.40)	0.939

BMI, body mass index; CI, confidence interval; OR, odds ratio.
Model 1: Adjusted for age and year
Model 2: Additionally adjusted for region, household income, parental education level, and the number of siblings
Model 3: Additionally adjusted for regular breakfast habits, frequency of eating out, and physical activity
Family structure categories indicate participants' living arrangements (ie, living with both parents, a single parent, only grandparents, or grandparents and parents).

eTable 2. Multivariable logistic regression analysis of overweight and obesity according to family structure among male adolescents

			BMI ≥85th		BMI ≥95th	
			OR (95% CI)	*P* value	OR (95% CI)	*P* value
10–14 years	Model 1	Both parents	1		1	
		Single parent	1.39 (0.90– 2.14)	0.142	1.12 (0.66–1.89)	0.673
		Only grandparents	4.38 (1.46–13.18)	0.009	2.06 (0.72–5.94)	0.180
		Grandparents and parents	2.26 (1.34–3.82)	0.002	2.27 (1.24–4.18)	0.008
	Model 2	Both parents	1		1	
		Single parent	1.37 (0.79–2.37)	0.264	1.19 (0.59-2.39)	0.632
		Only grandparents	4.61 (1.35–15.77)	0.015	2.06 (0.56–7.54)	0.275
		Grandparents and parents	2.36 (1.35–4.11)	0.002	2.25 (1.23–4.10)	0.008
	Model 3	Both parents	1		1	
		Single parent	1.60 (0.89–2.86)	0.115	1.21 (0.60–2.41)	0.597
		Only grandparents	5.41 (1.25–23.43)	0.024	2.29 (0.50–10.52)	0.286
		Grandparents and parents	2.48 (1.39–4.41)	0.002	2.42 (1.25–4.69)	0.009
15–18 years	Model 1	Both parents	1		1	
		Single parent	0.98 (0.56–1.70)	0.940	0.81 (0.40–1.65)	0.562
		Only grandparents	2.05 (0.43–9.81)	0.368	2.01 (0.35–11.68)	0.436
		Grandparents and parents	2.72 (1.27–5.85)	0.010	3.40 (1.41–8.19)	0.007
	Model 2	Both parents	1		1	
		Single parent	1.40 (0.70–2.81)	0.340	1.64 (0.66–4.11)	0.285
		Only grandparents	3.08 (0.49–19.19)	0.228	5.62 (0.66–48.17)	0.115
		Grandparents and parents	2.61 (1.23–5.54)	0.013	3.63 (1.57–8.36)	0.003
	Model 3	Both parents	1		1	
		Single parent	1.28 (0.57–2.86)	0.543	2.28 (0.75–6.91)	0.095
		Only grandparents	2.61 (0.37–18.49)	0.336	7.96 (0.62–102.29)	0.111
		Grandparents and parents	2.21 (0.96–5.12)	0.062	2.43 (1.02–5.76)	0.045

BMI, body mass index; CI, confidence interval; OR, odds ratio.
Model 1: Adjusted for age and year
Model 2: Additionally adjusted for region, household income, parental education level, and the number of siblings
Model 3: Additionally adjusted for regular breakfast habits and frequency of eating out
Family structure categories indicate participants' living arrangements (ie, living with both parents, a single parent, only grandparents, or grandparents and parents).

eTable 3. Multivariable logistic regression analysis of overweight and obesity according to family structure among female adolescents

			BMI ≥85th		BMI ≥95th	
			OR (95% CI)	*P* value	OR (95% CI)	*P* value
10–14 years	Model 1	Both parents	1		1	
		Single parent	1.04 (0.62–1.73)	0.886	0.67 (0.30–1.50)	0.331
		Only grandparents	2.09 (0.45–9.79)	0.350	2.39 (0.54–10.71)	0.253
		Grandparents and parents	1.67 (0.96–2.92)	0.070	1.35 (0.68–2.68)	0.397
	Model 2	Both parents	1		1	
		Single parent	1.05 (0.53–2.08)	0.892	0.54 (0.20–1.43)	0.215
		Only grandparents	2.81 (0.48–16.41)	0.252	1.78 (0.27–11.75)	0.549
		Grandparents and parents	1.60 (0.88–2.88)	0.120	1.06 (0.51–2.17)	0.883
	Model 3	Both parents	1		1	
		Single parent	0.96 (0.46–1.99)	0.902	0.54 (0.20–1.43)	0.214
		Only grandparents	2.95 (0.43–20.33)	0.271	1.56 (0.21–11.48)	0.663
		Grandparents and parents	1.49 (0.78–2.87)	0.228	0.89 (0.41–1.96)	0.775
15–18 years	Model 1	Both parents	1		1	
		Single parent	1.10 (0.65–1.89)	0.717	0.93 (0.48–1.82)	0.838
		Only grandparents	1.05 (0.07–16.41)	0.973	1.30 (0.09–18.45)	0.847
		Grandparents and parents	1.18 (0.50–2.76)	0.709	0.69 (0.22–2.20)	0.535
	Model 2	Both parents	1		1	
		Single parent	1.15 (0.56–2.38)	0.704	0.76 (0.34–1.70)	0.505
		Only grandparents	1.06 (0.05–23.58)	0.970	0.75 (0.04–15.64)	0.851
		Grandparents and parents	1.15 (0.48–2.77)	0.758	0.65 (0.19–2.15)	0.477
	Model 3	Both parents	1		1	
		Single parent	1.27 (0.59–2.76)	0.545	0.78 (0.33–1.85)	0.448
		Only grandparents	1.36 (0.05–39.74)	0.859	0.95 (0.03–31.10)	0.747
		Grandparents and parents	0.92 (0.33–2.61)	0.883	0.59 (0.14–2.46)	0.468

BMI, body mass index; CI, confidence interval; OR, odds ratio.
Model 1: Adjusted for age and year
Model 2: Additionally adjusted for region, household income, parental education level, and the number of siblings
Model 3: Additionally adjusted for regular breakfast habits and frequency of eating out
Family structure categories indicate participants' living arrangements (ie, living with both parents, a single parent, only grandparents, or grandparents and parents).
